# Determinants of high-risk fertility behavior among reproductive-age women in Ethiopia using the recent Ethiopian Demographic Health Survey: a multilevel analysis

**DOI:** 10.1186/s41182-020-00280-1

**Published:** 2020-11-27

**Authors:** Zemenu Tadesse Tessema, Koku Sisay Tamirat

**Affiliations:** Department of Epidemiology and Biostatistics, Institute of Public Health, College of Medicine and Health Sciences, University of Gondar, Gondar, Ethiopia

**Keywords:** High-risk fertility behavior, Determinants, Ethiopia, Reproductive-age women

## Abstract

**Background:**

High-risk fertility behavior is associated with numerous unfavorable child and maternal health outcomes such as chronic undernutrition, anemia, and child mortality. As far as our knowledge goes, there is not much study on determinants of high-risk fertility behavior in Ethiopia. Therefore, this study aimed to assess determinants of high-risk fertility behavior among reproductive-age women in Ethiopia.

**Method:**

The study was based on secondary data analysis from the 2016 Ethiopia Demography and Health Survey. A total of 11,022 women who gave birth 5 years preceding the survey were included in this study. Kid’s Record (KR) dataset was used. The adjusted odds ratio (AOR) with its 95% confidence interval (CI) was calculated for those variables included in the multilevel logistic regression model. *P* value ≤ 0.05 was employed to declare the statistically significant variables.

**Results:**

More than three-fourths (76.9%) of (95% CI 76.11 to 77.69) reproductive-age women had at least one high-risk fertility behavior. Attended primary and secondary education adjusted odds ratio (AOR) (AOR = 0.71; 95% CI 0.63, 0.80 and AOR = 0.73; 95% CI 0.60, 0.89, respectively), never used contraceptive (AOR = 1.25, 95% CI 1.12, 1.40), unwanted pregnancies (AOR = 1.40, 95% CI 1.23, 1.59), had no ANC visit (AOR = 1.19, 95% CI 1.05, 1.35), rural-dwelling (AOR = 1.26, 95% CI 1.04, 1.51), regions of Ethiopia [Somalia (AOR = 1.70; 95% CI 1.24, 2.32) and Amhara (AOR = 0.72; 95% CI 0.53, 0.96)] were determinants of high-risk fertility behavior.

**Conclusion:**

Education, rural residence, unwanted pregnancies, no antenatal care follow-up, and never used contraceptives were determinants of high-risk fertility behavior. Therefore, increased maternal health services, special intervention for hotspot areas, and giving special attention to rural dweller women were highly recommended.

## Background

Approximately 810 women die every day, from preventable causes related to pregnancy and childbirth according to a 2017 report. Between 2000 and 2017, the maternal mortality ratio (MMR, the number of maternal deaths per 100,000 live births) dropped by about 38% worldwide. Ninety-four percent of all maternal deaths occur in low and lower-middle-income countries. Young adolescents (ages 10–14) face a higher risk of complications and death as a result of pregnancy than other women [1]. By 2030, all countries should reduce the maternal mortality ratio (MMR) by at least two-thirds of their 2010 baseline level. The average global target is an MMR of less than 70/100,000 live births by 2030 [2]. It is currently under Sustainable Development Goal (SDG) 3 target 3.1: targeted to reduce below 70 deaths per 100,000 live births at the end of 2030 [2]. The supplementary national target is that no country should have an MMR greater than 140/100,000 live births (a number twice the global target) by 2030 [3]. Sub-Saharan Africa had the highest MMR in 2015, an estimated 546 maternal deaths per 100,000 live births [4]. Ethiopia is one of the countries with the highest maternal mortality ratio, with 412 deaths per 100,000 live births according to the 2016 EDHS reports, of which most of the deaths were attributed to high-risk fertility behavior [5, 6]. The global population is rapidly increasing, and according to the 2016 report, the total fertility was 2.5 and 4.8 per woman globally and in Ethiopia, respectively [7]. Fertility behavior of women is characterized by maternal age, birth spacing, and order, which has an impact on the health of women and children [8, 9]. Chronic undernutrition, anemia, and child and maternal mortality are unfavorable children and maternal health associated with high-risk fertility behavior [10–12]. Pieces of evidence from different works of the literature revealed that stillbirth, low birth weight, and prematurity were associated with high-risk fertility behavior [12–15]. The birth interval is another factor associated with high-risk fertility behavior. When birth interval got narrower, i.e., less than 24 months, the chance of child morality increased sharply compared to long-spaced birth intervals [16]. The infant born from teenage mothers increased the risk of mortality by 30% compared to their counterparts. The problem is higher in low-income countries where healthcare services are difficult to access and there are low socio-economic conditions and high magnitude of unmet family planning need [11, 13, 14, 17–20]. Also, early-age women marriage is another problem for high-risk fertility problems in Ethiopia and other low- and middle-income countries [18].

High-risk fertility behavior can be affected by socio-demographic characteristics; residence [21, 22], religion, level of education, and marital status are associated factors with high-risk fertility behavior. Similarly, reproductive health characteristics such as a history of child death, facility delivery, and family planning utilization are determinants of high-risk fertility behavior [10, 11, 13, 15, 18, 19, 23].

To our search and knowledge, there is a scarcity of information about determinants of high-risk fertility behavior in Ethiopia. Therefore, this study aimed to assess the determinants of high-risk fertility behavior among reproductive-age women in Ethiopia. The study will help health planners and policymakers to further reduce high-risk fertility behavior in Ethiopia and provide baseline information to other researchers.

## Methods

### Study design, area, and period

A population-based cross-sectional study design was used on the Ethiopian Demographic and Health Survey (EDHS) 2016 datasets. Ethiopia is situated in the Horn of Africa. It has a total area of 1,100,000 km^2^ and lies between latitudes 3° and 14° N, and longitudes 33° and 48° E. It has 9 regional states (Afar; Amhara; Benishangul-Gumuz; Gambela; Harari; Oromia; Somali; Southern Nations, Nationalities, and People’s Region (SNNP); and Tigray) and two administrative cities (Addis Ababa and Dire Dawa). The data was collected from January 18, 2016, to June 27, 2016.

### Data source and study population

The data source was EDHS 2016 IR (Individual Records) dataset. The source population was all women age 15–49 years in the enumeration areas within 5 years before the survey in Ethiopia. 15,683 women aged 15–49 years were interviewed, and a weighted sample of 11,023 women was included in the study. In the 2016 EDHS, a total of 645 clusters (EAs) (202 urban and 443 rural) were selected with a probability proportional to each EAs size and independent selection in each sampling stratum. The recorded data was accessed at www.measuredhs.com on request with the help of ICF International, Inc.

### Data collection tools and procedures

Ethiopian Demographic and Health Survey data were collected by two-stage stratified sampling. Each region of the country was stratified into urban and rural areas, yielding 21 sampling strata. In the first stage, 645 EAs were selected with a probability method proportional to the enumeration area size by independent selection in each sampling stratum. In the second stage of selection, a fixed number of 28 households per cluster were selected with an equal probability, systematic sampling from the newly created household listings. The detailed sampling procedure was available in the Ethiopian Demographic and Health Survey reports from Measure DHS website (www.dhsprogram.com) [6].

### Outcome variable

For this study, we considered three parameters, maternal age at the time of delivery, birth order, and birth interval, to define the high-risk fertility behaviors. Three exposure variables were defined for this analysis. Any high-risk fertility behavior versus non-risk is coded as 1/0, respectively. The presence of any of the following four conditions was termed high-risk fertility behavior (coded as 1 and otherwise 0): mothers aged less than 18 years at the time of delivery, mothers aged over 34 years at the time of delivery, the latest child born less than 24 months after the previous birth, and latest child of order three or higher. We applied the definition of “high-risk fertility behaviors” adopted by the 2016 EDHS [6].

### Independent variables

Based on different literature, independent variables for this study were demographic characteristics such as sex of a child, religion, educational level, occupation, wealth index, media exposure, residence and region, and reproductive characteristics such as contraceptive use, wanted pregnancy, ANC follow-up, stillbirth, and place of delivery [10, 11, 13, 15, 18, 19, 23].

### Data processing and analysis

After data was cleaned and extracted, descriptive statistics and multilevel logistic regression analysis were done using STATA version 14.1. The data were weighted using cluster number, primary sampling unit, and strata before any statistical analysis to restore the representativeness of the survey and to tell the STATA to consider the sampling design when calculating SEs.

### Model building

We fitted four models, the null model without predictors, model I with only individual-level variables, model II with only community-level variables, and model IV both individual-level and community-level variables. These models were fitted by a STATA command “xtmelogit” to identify predictors of the outcome variable. For model comparison, we used the log-likelihood ratio (LLR) and Akaike Information Criteria (AIC) tests. The highest log-likelihood and the lowest AIC win the best-fitted model.

### Parameter estimation methods

In the multilevel logistic regression model, fixed effects estimates measure the association between the odds of high-risk fertility behavior (HRFB) of individual- and community-level factors with a 95% confidence interval. The random effect measures variation HRFB across clusters expressed by intraclass correlation (ICC), quantifies the degree of heterogeneity of HRFB between clusters [24]; percentage change in variance (PCV), the proportion of the total observed individual variation in the HRFB that is attributable by cluster variations [25]; and median odds ratio (MOR), median value of the odds ratio between the cluster at HRFB and cluster at lower risk of HRFB when randomly picking out two clusters (EAs) [26].

### Ethics approval and consent to participate

Ethical clearance was obtained from measure DHS through filling a form requesting for accessing data. The data used in this study are publicly available, aggregated secondary data with no personal identifying information that can be linked to study participants. The confidentiality of the data was maintained anonymously.

## Result

### Socio-demographic characteristics of respondents

A total of 11,022 women who gave birth in the preceding 5 years before the survey were included in the final analysis. The median age of women was 28, with an interquartile range of 25 to 34 years; about half (53%) aged between 25 and 34. The majority (89%) of women were rural dwellers, most (93.1%) were married, and about 41.4% and 34.2% were Muslim religion and Orthodox religion follower, respectively. Two-thirds (66.1%) of the women had not attended any formal education, and 59.3% had no occupation (Table 1).

**Table 1:** Sociodemographic characteristics of women who gave birth in the preceding 5 years before the survey in Ethiopia, 2016 (*n* = 11,022)

Variable	High-risk fertility problem		Total (%)	X-square value	***p*** value
	Yes	No			
**Residence**					
Rural	808	407	1215 (11.03)	81.55	< 0.001
Urban	7809	2197	9807 (88.97)		
**Age group**					
15–24	1779	666	2445 (22.19)	644.08	< 0.001
25–34	4025	1817	5842 (53.00)		
35–49	121	2613	2734 (24.81)		
**Religion**					
Orthodox	2231	851	3082 (34.22)	87.82	< 0.001
Muslim	1048	4394	5442 (41.38)		
Protestant	1385	477	1862 (22.13)		
Others	200	55	255 (3.27)		
**Maternal education**					
No education	5527	1311	6837 (66.08)	152.37	< 0.001
Primary education	1918	760	2678 (26.77)		
Secondary	500	234	734 (4.66)		
Higher	265	126	391(2.49)		
**Marital status**					
Married	7833	2423	10257 (93.1)	111	< 0.001
Single	284	481	765 (6.90)		
**Husband education**					
No education	3965	1090	5076 (47.82)	235	< 0.001
Primary education	3103	1012	4115 (39.34)		
Secondary	572	226	798 (7.63)		
Higher	314	156	471 (4.05)		
**Maternal occupation**					
Had occupation	3715	1180	4895 (44.42)	9.85	0.002
Had no occupation	4702	1424	6126 (55.58)		
**Wealth index**					
Poor	4049	1106	5155 (46.78)	89.67	< 0.001
Middle	1736	542	2279 (20.68)		
Rich	2630	955	3586 (32.54)		
**Media exposed**					
Yes	622	304	987(8.96)	88.27	< 0.001
No	7794	2240	10035(91.04)		
**Sex of child**					
Male	4404	1320	5724(51.94)	1.62	0.202
Female	4013	1284	5297(48.06)		

### Reproductive characteristics and high-risk fertility behaviors

This study revealed that 76.5% (95% CI 75.1 to 77.1) of women had high-risk fertility behavior, of which 31.4% were in single-risk category, 27.1% of them had a birth interval of fewer than 24 months, and about 45.1% of women were categorized in multiple high-risk groups.

Out of total mothers who participated, 43.1% of them were grand multiparous; the majority (75%) of pregnancy for the recent births were wanted, of which three-fourth (74.4%) mothers had antenatal care follow-up for their last child. The majority (98.1%) of women gave birth vaginally for their recent child, 72.5% delivered at home, and 8.5% had a stillbirth. About 30.1% of women had anemia during pregnancy (Table 2).

**Table 2:** Reproductive and high-risk fertility behavior of women who gave birth 5 years before the survey in Ethiopia, 2016 (*n* = 11,022)

Variable	High-risk fertility problem		Total (%)	X-square value	***p*** value
	Yes	No			
**Wanted pregnancy**					
Yes	6189	2089	8279 (75.11)	7.89	0.005
No	2228	514	2743 (24.89)		
**Place delivery**					
Home	6234	1762	7997 (72.55)	68.80	< 0.001
Health institution	2183	842	3025 (27.45)		
**History of stillbirth**					
Yes	748	217	966 (8.77)	1.11	0.291
No	7668	2387	10,056 (91.23)		
**Anemia**					
Severe	134	22	157 (1.48)	31.59	< 0.001
Moderate	694	150	744 (7.00)		
Mild	1833	480	2321 (21.82)		
No anemia	5562	1853	7416 (69.70)		
**Birth order**					
1	2057	1	2058 (18.62)	1.1	0.412
2–4	2113	2604	4718 (42.81)		
5+	4241	3	4245 (38.52)		
**Parity**					
Primiparous	1433	1	1434 (13.01)	1.5	0.123
Multiparous	2266	2568	4833 (43.87)		
Grand multiparity	4716	35	4752 (42.1)		
**Delivery type**					
Vaginal	8277	2532	212 (1.9)	6.41	0.011
Cesarean	140	72	10,909 (98.1)		

### Prevalence of high risk fertility behavior

**Fig. 1:**
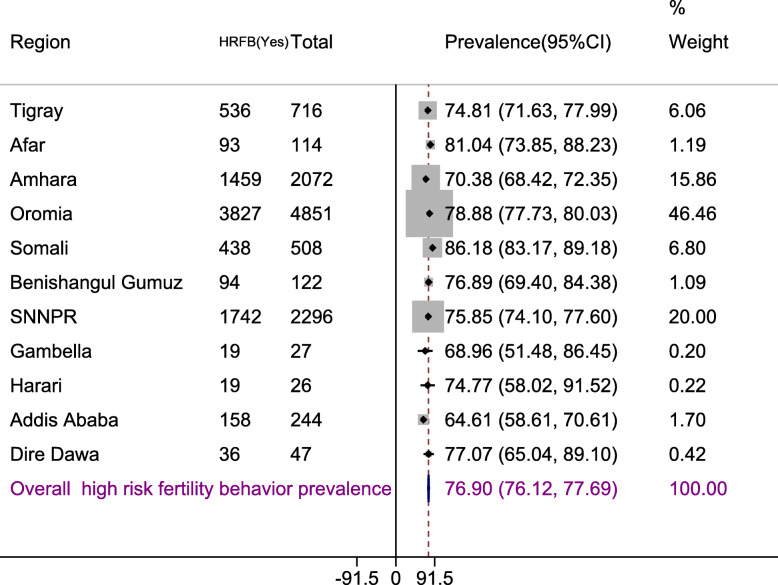
Regional distribution of high-risk fertility behavior in Ethiopia

The prevalence of high-risk fertility behavior in Ethiopia was 76.90% with 95% confidence interval 76.12 to 77.69%. The higher high-risk fertility (86.18%) was detected in Somalia, and the smaller high-risk fertility behavior (64.61%) was detected in Addis Ababa (Fig. 1)

### Individual- and community-level determinants of high-risk fertility behavior

The results of multilevel logistic regression for the individual-level and community-level variables are presented in Table 3. In the full model in which all individual-level and community-level factors included the education status of women, ANC visits during pregnancy, contraceptive utilization, wanted pregnancy, residence, and region were elements that were significantly associated with high-risk fertility behavior.

**Table 3:** Multilevel logistic regression analysis of high-risk fertility behavior among reproductive-age women in Ethiopia, 2016 (*n* = 11,022)

Variables	Category	Model (I) AOR (95% CI) without factor	Model (II) AOR (95% CI) individual-level factor	Model (III) AOR (95% CI) community-level factor	Model (IV) AOR (95% CI) individual + community-level factor
Sex of child	Male		Ref		Ref
	Female		0.94 (0.85, 1.03)		0.93 (0.85, 1.02)
Religion	Orthodox		Ref		Ref
	Muslim		1.35 (1.19, 1.52)		1.10 (0.93, 1.29)
	Protestant		1.07 (0.93, 1.24)		1.08 (0.89, 1.30)
	Others		1.14 (0.82, 1.59)		1.15 (0.82, 1.62)
Educational status of mother	Unable to read and write		Ref		Ref
	Primary education		0.69 (0.62, 0.78)		0.71 (0.63, 0.80)*
	Secondary education		0.69 (0.57, 0.84)		0.73 (0.60, 0.89)*
	Higher education		0.76 (0.59, 0.99)		0.82 (0.63, 1.07)
Occupational status women (the mother)	Working		Ref		Ref
	Not working		0.99 (0.89, 1.09)		0.96 (0.87, 1.06)
Wealth index	Poor		Ref		Ref
	Middle		0.90 (0.78, 1.04)		0.95 (0.82, 1.10)
	Rich		0.91 (0.80, 1.04)		0.98 (0.85, 1.13)
Media exposure	Has media exposure		Ref		Ref
	No media exposure		0.81 (0.69, 0.96)		0.83(0.70,1.004)
Contraceptive use	Yes		Ref		Ref
	No		1.31 (1.17, 1.47)		1.25 (1.12, 1.40)*
Wanted pregnancy	Yes		Ref		Ref
	No		1.34 (1.18, 1.53)		1.40 (1.23, 1.59)*
Had ANC	Yes		Ref		Ref
	No		1.19 (1.05, 1.34)		1.19 (1.05, 1.35)*
Stillbirth	No		Ref		Ref
	Yes		1.08 (0.88, 1.28)		1.07 (0.90, 1.27)
Delivery place	Institution		Ref		Ref
	Home		0.99 (0.88, 1.12)		0.97 (0.85, 1.11)
Residence	Urban			Ref	Ref
	Rural			1.65 (1.43, 1.90)	1.26 (1.04, 1.51)*
Region	Addis			Ref	Ref
	Afar			1.56 (1.16, 2.10)	1.19 (0.86, 1.63)
	Amhara			0.81 (0.61,1.08)	0.72 (0.53, 0.96)*
	Oromia			1.18 (0.89, 1.57) 2.289	0.98 (0.73, 1.31)
	Somalia			2.29 (1.73, 3.05)	1.70 (1.24, 2.32)*
	Benishangul Gumuz			1.12 (0.83, 1.52)	0.99 (0.73, 1.35)
	SNNP			1.05 (0.79, 1.39)	0.93 (0.69, 1.26)
	Gambela			0.92 (0.69, 1.24)	0.79 (0.57, 1.09)
	Harari			1.08 (0.80, 1.47)	0.97 (0.71, 1.32)
	Tigray			1.07 (0.811, 1.43)	1.03 (0.77, 1.39)
	Dire Dawa			1.43 (1.05, 1.94)	1.22 (0.88, 1.68)
**Random effects (effects of variation), i.e, measure of variation for high-risk fertility behavior**					
Community level variance(SE)		0.188 (0.032)	0.062 (0.024)	0.064 (0.023)	0.039 (0.024)
*p* value		< 0.001	< 0.001	< 0.001	< 0.001
Deviance		11,372	11,146	11,202	11,092
ICC%		5.4	1.8	1.91	1.18
PCV%		Reference	67.03	65.97	79.26
MOR		1.50	1.26	1.27	1.20

*Ref* reference category

*p value less than 0.05

Women who attended primary or secondary education at odds of high-risk fertility behavior decreased by 29 and 27%, respectively, compared to those who had no formal education (AOR = 0.71; 95% CI 0.63 to 0.80 and AOR = 0.73; 95% CI 0.60 to 0.89). For those women who had not used contraceptives previously, the odds of high-risk fertility behavior were increased by 25% compared to those who had used contraceptives (AOR = 1.25; 95% CI 1.12 to 1.40). For those women who had unwanted pregnancies, the odds of high-risk fertility behavior were 40% more likely than those who had wanted pregnancies (AOR = 1.40; 95% CI 1.23 to 1.59). For women who had not to use antenatal care visits for their recent child, the odds of high-risk fertility behavior were 19% more likely than those who had follow-ups (AOR = 1.19; 95% CI 1.05 to 1.35).

Moreover, living in a rural area was also associated with 26% increased odds of high-risk fertility behavior among women of reproductive age than urban residents (AOR = 1.26; 95% CI 1.04 to 1.51). Higher odds of high-risk fertility behavior were observed in the Somali regional state (AOR = 1.70; 95% CI 1.24 to 2.32) compared with Addis Ababa. However, the odds of high-risk fertility behavior among women were lower in the Amhara region compared with Addis Ababa (AOR = 0.72; 95% CI 0.53 to 0.96) (Table 3).

The high-risk fertility behavior prevalence rate was not similarly distributed across the communities. About 5.4% of the variance in the odds of high-risk fertility behavior in women could be attributed to community-level factors, as calculated by the ICC based on estimated intercept component variance. The variation was also statistically significant (*p* value < 0.001). After adjusting for individual-level and community-level factors, the variation in high-risk fertility behavior across communities remained statistically significant. About 1.18% of the odds of high-risk fertility behavior variation across communities were observed in the full model (model 4).

Moreover, the MOR indicated that high-risk fertility behavior was attributed to the community-level factors. The MOR for high-risk fertility behavior was 1.50 in the empty model (model 1), which showed that there were variations between communities (clustering) since MOR was 1.5 times higher than the reference (MOR = 1). The unexplained community variation in high-risk fertility behavior decreased to MOR of 1.20 when all factors were added to the null model (empty model). This indicates that when all elements were included, the clustering effect is still statistically significant in the full model (Table 3).

## Discussion

This study revealed that more than three-fourths of women had high-risk fertility behavior of 31.4% and 45.1% in single and multiple risk categories, respectively. The birth interval of fewer than 24 months was the most common single risk on 27.4% of women. This finding was lower than a study conducted in the Afar region of Ethiopia (86.3%) [27]. However, this finding was higher than 58% of 2011 EDHS report [28], 34% in Bangladesh DHS [29], 38.3% in Nepal [30], and 44.9% in India [29]. The possible explanation for the observed discrepancies might be because of socio-demographic characteristic changes and increased intention of fertility in society. Specifically, compared to Asian countries such as Nepal, the socio-demographic characteristics are quite different, and the health system variations could also be the reason. In Ethiopia, child marriage is higher, which might be responsible for the increased magnitude of risky fertility behaviors [30]. Particularly, this finding was higher than the 2011 EDHS report of 58%, which could be due to the reasons for increased fertility intention.

Those women who attended primary and secondary school associated with a decreased probability of high-risk fertility behavior compared to those with no formal education. This finding was consistent with the study result in Ethiopia, Nigeria, and Nepal [9, 28, 29, 31]. This could be because those attending school had better knowledge and awareness about high-risk fertility behavior and lower probability of experiencing early marriage.

Women who never used contraceptive methods previously associated with an increased occurrence of high-risk fertility behavior compared to those who had used it. This finding was supported by other studies and evidence [18, 20]. One of the purposes of contraceptive use is spacing birth intervals and decreasing unintended pregnancies, which might affect the mother and child’s health; thus, the essential postnatal services intended to widen birth intervals through the provision of family planning services [23].

Women who had a history of unwanted pregnancies were more likely to had high-risk fertility behavior than wanted pregnancies. The possible reason might be women who had experienced unwanted pregnancies is an indicator of low family planning utilization. This finding was consistent with other studies conducted in Nigeria [9].

Women who were rural dwellers have increased odds of high-risk fertility behavior compared to those urban dwellers. This finding was consistent with the study conducted in Ethiopia [28]. The possible reason might be women in rural areas are highly disadvantaged in terms of reproductive health services besides low literacy levels in rural areas. Those women who had no ANC follow-ups to the recent children were associated with increased risky fertility behaviors. During ANC, follow-up clinical checkups were made for the mother and fetus. Furthermore, counseling about postnatal care included family planning choices for the widening of intervals between births. Thus, the low utilization of ANC during pregnancy might contribute to high-risk fertility behavior.

Compared to Addis Ababa women who reside in the Somalia region, the high-risk fertility behaviors were doubled. In contrast, for women who live in the Amhara region, high-risk fertility behavior were decreased. This might be explained by health service inaccessibility and low family planning acceptance rates due to community beliefs and myths from religious perspectives. Moreover, in the Somali region, the community follows nomadic ways of life and has difficulty with health services in addition to a serious security problem.

This study has strengths of the data being nationally representative, the multilevel analysis used to account cluster correlations. However, this study has faced the following limitations: firstly, the study’s cross-sectional nature affects the cause-effect relationship. Secondly, health system characteristics were not assessed; lastly, the data of this study had problems of recall bias, such as several months for the birth interval.

## Conclusion

In this study, high-risk fertility behavior was high with significant regional variations. Education, rural residence, unwanted pregnancies, no antenatal care follow-up, and never used contraceptives were determinants of high-risk fertility behavior. Therefore, increased maternal health services, special intervention for hotspot areas, and giving special attention to rural dweller women were highly recommended.

## Data Availability

The datasets used during the current study are available from the corresponding author

## Abbreviations

ANC: Antenatal care
AOR: Adjusted odds ratio
CI: Confidence interval
DHS: Demographic and health survey
EA: Enumeration area
E: East
EDHS: Ethiopian Demographic and Health Survey
ICC: Intraclass correlation
KR: Kid’s Record
LLR: Likelihood ratio
MOR: Median odds ratio
N: North
PCV: Proportion of cluster variance
SE: Standard error
SGD: Sustainable development goal
SNNP: Southern Nations, Nationalities, and People’s Region
